# Doomed drones? Using passage experiments and mathematical modelling to determine Deformed wing virus population dynamics in male honeybees

**DOI:** 10.1098/rspb.2023.1010

**Published:** 2023-06-28

**Authors:** Luke Woodford, Pieter C. Steketee, David J. Evans

**Affiliations:** ^1^ Department of Biology, University of St. Andrews, Biomedical Sciences Research Complex, St. Andrews, None KY16 9ST, UK; ^2^ The Roslin Institute, Easter Bush Campus, Midlothian, Edinburgh, EH25 9RG, UK

**Keywords:** honeybee, drone, varroa, DWV, mathematical model

## Abstract

*Varroa destructor* is an ectoparasitic mite of honeybees which vectors a range of pathogenic viruses, the most notable being Deformed wing virus (DWV). Mites parasitise bees during pupal development and male honeybees, drones, have a longer development cycle than female workers (24 versus 21 days), allow for more progeny mites to develop per foundress (1.6–2.5 compared to 0.7–1.45). How this longer exposure time influences evolution of the transmitted virus population is unknown. Using uniquely tagged viruses recovered from cDNA we investigated the replication, competition and morbidity of DWV genotypes in drones. Assays examining virus replication and morbidity revealed drones are highly susceptible to both predominant genotypes of DWV. In virus passage studies using an equimolar inocula of major DNA genotypes and their recombinants, the recombinant form dominated but did not reach 100% of the virus population within 10 passages. Using an *in-silico* model of the virus–mite–bee system we examined bottlenecks during virus acquisition by the mite and subsequent injection of viruses into the host, which may play a significant role in shaping virus diversity. This study furthers our understanding of the variables influencing DWV diversity changes and provides insight into areas of future research in the mite–virus–bee system.

## 1. Introduction

Deformed wing virus (DWV) is an endemic single stranded positive sense RNA virus of honeybees which is transmitted by the ectoparasitic mite *Varroa destructor*. Together, *Varroa* and DWV are the most prevalent honeybee pathogens and are highly associated with global colony losses [1], in particular overwinter in temperate regions. In the absence of Varroa, DWV is typically spread horizontally in the colony via larval feeding or trophallaxis [2], and vertically via eggs and sperm [3,4]. Varroa alters the transmission route of DWV, by injecting the virus while feeding on developing pupae. As a consequence the virus replicates to very high levels in the recipient pupae, with many exhibiting developmental symptoms that result in death pre- or shortly post-eclosion. Bees emerging without overt developmental symptoms exhibit learning and memory defects and reduced longevity [5–8]. At the colony level, high mite levels threaten colony overwintering survival, primarily due to this reduced longevity and consequent the loss of workers [9].

*Varroa* have evolved to preferentially enter drone cells—up to 11.6 times more frequently—when they invade colonies [10]. The increased developmental time of drones (24 days versus 21 days for workers) allows the production of additional progeny mites (1.6–2.5 daughter mites in drone cells versus 0.7–1.45 daughter mites in worker brood cells) [11]. Despite this known preference for drone pupae, the majority of studies have focused on the consequences of mite-transmitted DWV on female workers rather than male drones.

There is a correlation between the level of mite infestation and an increase in the amount of DWV, but a decrease in the diversity of DWV at the colony level [12]. This has been inferred to mean that viruses preferentially amplified after mite transmission end up dominating the virus population, reflected in a decrease in diversity. However, more recent research has indicated that individual bees infected with high titres of DWV often contain diverse virus populations [13–15] and the factors that determine why a population retains or loses diversity is unknown. One possibility is that particular virus genotypes may have a replicative advantage; there are two major genotypes (A and B) which also freely recombine [16–18]. Various studies investigating serial passaging of viruses in worker bees have shown little change in the population across multiple generations. Yanez *et al.* [19] demonstrated a uniformity of the Type A virus population despite multiple generations of pupal injections and regardless of whether the eclosed bees were visibly symptomatic. Both Type A and B were shown to be present in equal abundance over five *in vitro* passages [20]. This may imply that the process of virus injection and replication in the host may be the same for all genotypes or that other processes, not replicated during *in vitro* studies (e.g. localized immune responses at the site of inoculation or virus replication in the mite vector), may be a significant influence on the resulting virus population.

If certain DWV genotypes replicated in the mite vector they may gain a selection advantage in the virus population. Contrasting reports have shown evidence of DWV replication in mites, depending on the variants investigated, with evidence of Type B and recombinants (encoding Type B structural proteins and Type A non-structural proteins) replicating in mites [21,22], whereas Type A variants are reported to be transmitted by mites in a non-propagative manner [23]. Given these findings and the difficulty in recapitulating the process of mite feeding and injection in the laboratory, even subtle differences in virus replication or transmissibility may, over time, influence the colony-level virus population. In other virus-vector systems, bottlenecks which shape the virus population have been defined. For example, in mosquito-vectored dengue virus as few as 5–42 infectious virus particles are ingested by the mosquito, resulting in a distinct bias in variants within the population that are subsequently mosquito-transmitted [24]. Similarly, Venezuelan equine encephalitis virus (VEEV) replicons infect an average of 28 midgut cells in the mosquito, likely contributing to a stringent virus bottleneck, similar to that observed in Dengue virus [25], and inoculated aphid vectors imposed a transmission bottleneck of 0.5–3.2 virions on potato Y virus [26]. A similarly stringent bottleneck occurring when mites feed on developing pupae may shape the virus population in honeybees, and therefore, greatly impact virus diversity.

In this study, we investigate the replication kinetics of clonal DWV populations, and demonstrate that all major DWV genotypes and recombinants achieve similar high viral loads in drones. In addition, the variants exhibit broadly similar morbidity rates and replicate to similar levels in coinfection experiments, as previously shown for worker pupae [21,27]. In serial passage experiments we find that a recombinant virus appears to have a replicative advantage that dominates the resulting virus population, though it does not result in 100% of the viruses within 10 passages. By applying a mathematical model to explore DWV replication kinetics, we predict that virus ingestion by the mite during pupal feeding has the most dramatic impact on virus population diversity, providing important insights on how selective advantages in DWV may arise over time in honeybee colonies. These data further our understanding of DWV population dynamics and the evolution of dominant variants.

## 2. Material and methods

### (a) Honey bees

All honeybees (*Apis mellifera*) used in this study were obtained from the University of St Andrews research apiary. Colonies were regularly and appropriately treated with miticides to manage Varroa levels and endogenous DWV levels were tested throughout the season. All pupae were removed from the comb and placed in an incubator 24 h prior to any injections.

### (b) Preparation of virus stocks

The infectious cDNA copies of DWV, designated DDD, VVV and VVD, have been described previously [21,28,29] and represent Type A, Type B and a recombinant (encoding Type B structural proteins and Type A non-structural proteins) of DWV respectively. Full sequences are available via GenBank accession numbers: DWV-DDD—MT415949; DWV-VVD (VVDH)—MT415950; and DWV-VVV—MT415952. All plasmid sequences were verified by Sanger sequencing. From these plasmids RNA was synthesized *in vitro* using a T7 Ribomax Express system (Promega). DWV was recovered following direct injection of honeybee pupae as described previously [28,29]. Crude viral stocks were obtained by homogenizing the injected bees in PBS and filtering the stock through a 0.22 µM filter before treating with DNase. The filtered stocks were quantified as described below.

### (c) Pupal injections, RNA quantification and PCR analysis

For infectivity and morbidity studies pink eyed pupae were injected using an insulin syringe (BD Micro Fine Plus, 0.3 ml, 30 G, Becton Dickinson) with crude viral stocks, diluted in PBS to the concentrations of 10^3^/10^6^ GE for infectivity and 10^2^ GE for morbidity, and incubated (30°C, 70% humidity) for 48 h for the infectivity experiment, or until eclosion for the morbidity experiment. All bees were analysed in the morbidity experiment, including those that died prior to eclosion.

Total RNA was subsequently extracted following tissue homogenization (Precellys Evolution tissue homogenizer; Stretton Scientific) with a Genejet RNA extraction kit (Thermo Fisher) according to manufacturer's instructions. Approximately 1 µg of RNA per sample was used to generate approximately 1 µg cDNA following the manufacturer's protocol (Quanta scientific) with both oligo dT and random hexamer primers included in the reaction mixture. qPCR reactions were performed for DWV in a CFX96 Touch Real-Time PCR Detection System (Bio-Rad). Reactions consisting of 1× Luna Universal qRT-PCR master mix (New England Biolabs), 0.25 µM forward and reverse primers and approximately 100 ng of total cDNA in a final volume of 20 µl. Type A and B variants were quantified using primer pairs unique to ‘Type A’ variants (DDD qPCR FP– 5′ GTCAAGAAGCAGGCGAATGTA 3′/RP – 5′ GCATAGGGGATCTAGAACACATAG 3′) and ‘Type B’ variants (VVV qPCR FP—5′ GAAAAGACGCGGGTGAGTTCG 3'/RP—5′ AACATTGGCGATCGATTACAAACG 3′) as per [28]. Negative template controls and a serial dilution of a positive control standard were included in each run. RT-qPCR standards were calculated from the standard curve generated from a serial dilution of each cDNA clone obtained from 1 µg of DWV DDD (for Type A) and VVV (for Type B) RNA transcript as previously described [28], with a linear range of 10^3^–10^10^ genomic equivalents (GE) per μg. The VVV and VVD constructs could not be distinguished by these primer sets, so are not co-infected in the infectivity or morbidity studies

### (d) Analysis of virus diversity after serial passage in drone pupae

**Figure 1.**
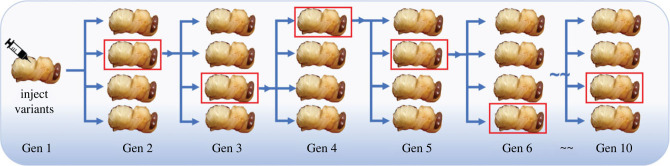
Serial passage schematic. Initial pupa injected with standardized mix of 3 variants, crude extracted virus stock was then injected into four pupae in each set with one pupa randomly selected for the next generation. This was performed in four replicates giving a total of 16 pupae injected in each generation. The pupae highlighted with red boxes were selected for the next generations injection and the ones subsequently analysed by NGS methods. Four generation of pupae were injected and analysed by RT-qPCR, but only three were selected for NGS analysis due to cost.

An equimolar mixed stock of VVV, DDD and VVD virus was prepared in PBS and diluted to 10^4^ GE/μg RNA per 5 µl. For passage experiments pink eyed drone pupae were injected with 5 µl of the virus mix and incubated for 48 h. In each case the virus was injected into the first pupa and left for 16 h to create a ‘stock’ population of viruses (Gen 1 in figure 1) to mimic the low-level DWV population found in honeybees with no mite exposure (typically 10^4^–10^6^ GE/μg; [15,16]). Virus preparations were then extracted from individual pupae to obtain a stock to inject the next generation. Virus extraction involved the homogenisation of pupae in 500 µl of PBS, centrifugation at 4000 g, 4°C for 10 min and passing the supernatant through a 0.22 µM filter. 400 µl of this stock extract was subsequently used for RNA preparation and purification prior to analysis by RT-qPCR for virus quantification (see above). An aliquot of the remaining stock was diluted 1/100 in PBS and injected into the next generation of pupae. This process was repeated for 10 generations, with one pupa selected at random from each group as the stock for the next generation as shown in figure 1. The same process was performed with a control set, whereby the first injection was PBS only and all future generations were injected with homogenized and filtered extract from the generation before. For the virus injected samples, in each generation 4 stocks were prepared, and 4 bees were then injected with each stock (total 16 per generation). Therefore, 4 unique virus passages were performed across ten generations of pupae. Three of these four passages were then analysed by next generation sequencing.

To analyse the virus population in each of the ten pupal generations, samples were prepared for next generation sequencing. One bee per generation in each of three of the four biological replicates was selected for sequencing, giving a total of 30 samples across the ten passaged generations (see electronic supplementary material, data files for sample selection). The three infectious clones could be distinguished by their sequence from one another and from any pre-existing infecting virus by the inclusion of translationally silent restriction sites. A region of approximately 1500 bp encompassing a known hotspot of recombination (a region spanning the junction encoding the structural and non-structural proteins) and the introduced silent restriction site tags (AvrII and HpaI as per [23]) was amplified using primer pair FP 5′ ATTGATCATTGTATGTTTACCTTCCCTTG 3′ and RP 5′ GCACGTAAGAGCTCGCTGCATA 3′ with hot start *Taq* polymerase under the following conditions: an initial denaturation step of 94°C for 20 s, 30 cycles of 94°C for 15 s, 57°C for 20 s and 68°C for 2 min, and a final extension at 68°C for 10 min. Amplicons were purified and each sample was barcoded for Illumina Miseq sequencing (data available at NCBI BioProject: PRJNA888084). Illumina reads were converted to fasta format, extracted and trimmed using Geneious Prime (v. 2019.1.3). To determine the proportions of each of the three variants present in any generation the haplotype diversity of the sequenced samples was determined using ShoRAH (*sho*rt *r*ead *a*ssembly into *h*aplotypes) [15,30,31]. All settings were run as per the defaults with ShoRAH, using the amplicon protocol and all haplotypes constituting greater than 1.5% of the viral population were included in the final population diversity analysis, based on the positive control samples analysed in this study. Two control samples were sequenced, an equimolar mix of the three clones pre-PCR amplification, and a post-PCR mix determined by purification (Promega Wizard SV kit, UK) and nanodrop of the three PCR products (figure 4*a*).

### (e) An in-silico model of virus population changes in a bee/mite/virus system

**Figure 2.**
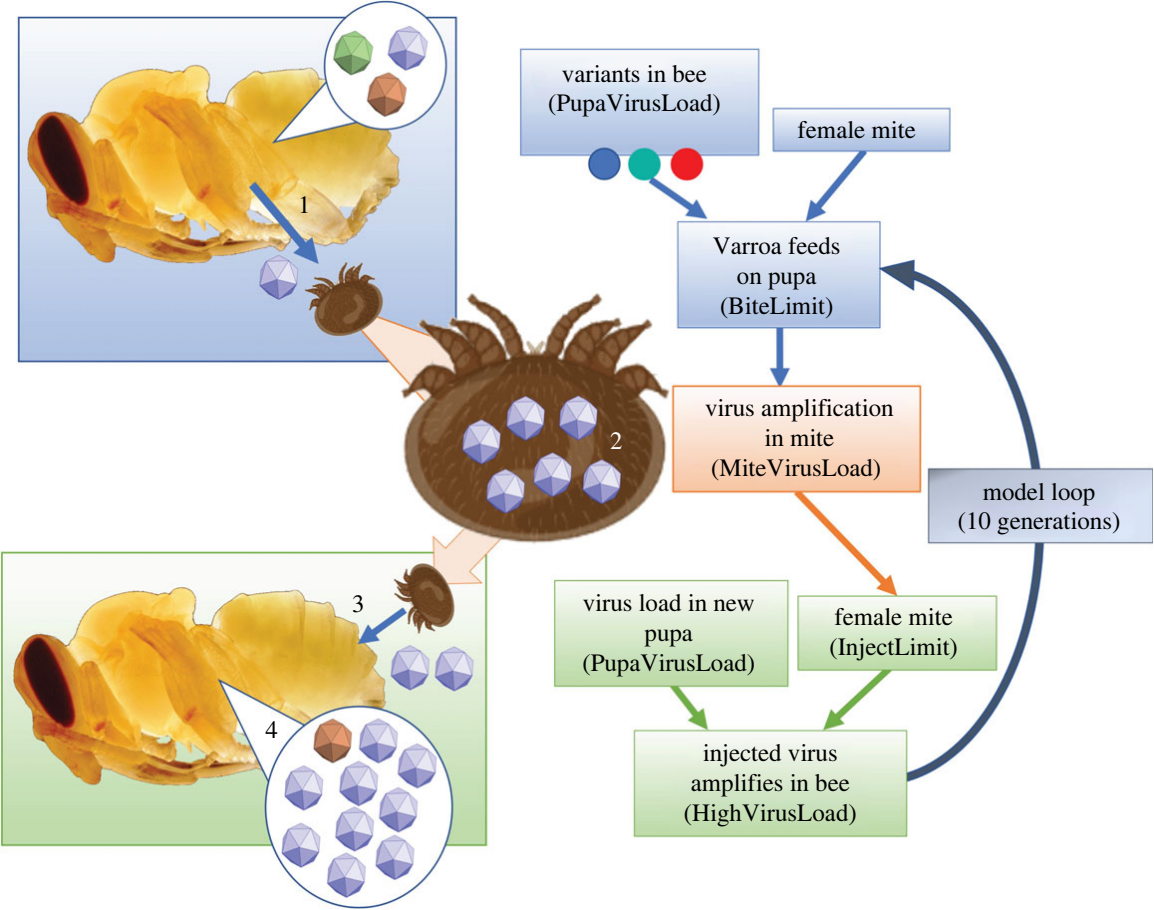
Schematic of the mite/bee/virus model. Showing the initial pupa with a mixed virus population, the mite feeding and acquiring virus, virus replication in the mite and subsequent injection of viruses into a pupa.

The model to measure potential bottleneck events in the virus population was generated in R studio (V3) [32]. Variables were determined based on published data and evidence from experimental analysis (table 1) and some of the variables could be adjusted across a stated range, for example the virus acquisition could be changed to any level between 5–50 viruses. Each variable was defined as per table 2 and could be altered for each run, depending on the variable being examined. The model is illustrated graphically in figure 2 and the full script is available in the electronic supplementary material, files.

**Table 1.** Parameters of the mite model. Each parameter in the model, whether that parameter is fixed or variable and where the information for the criteria originates from. The number in parenthesis in the left column indicates where each parameter occurs in the schematic shown in figure 2.

parameter	fixed or variable	rationale
viruses in bee	1000	based on RT-qPCR analysis of healthy bees [15,16]
virus diversity in bee	3 variants	DDD / VVV / VVD used in passage experiment
Varroa virus acquisition (1)	5–50	5–42 dengue copies in mosquitões [24]
virus amplification in mite (2)	10^4^–10^7^	RT-qPCR data [33]
virus injection by mite (3)	10^2^–10^5^	0.1–100% of virus amplification in mite [33]
virus amplification in next bee (4)	10^6^–10^9^	based on pupal injections [21]

**Table 2.** Variables contained in the model. Before starting the for-loop, the following variables were defined. These variables were determined and fixed for the full 10 cycles of each run, except the last two which were contained within the for loop and could therefore be randomized in every cycle or fixed at a set value.

model variable	value	default value	description
PupaVirusLoad	1000	1000	the number of viruses in the first pupa
Variants	A/B/C	A/B/C	variants, based on experiments with infectious clones
BiteLimit	5–50	10	maximum number of viruses mite takes during a bite/feed
MiteVirusLoad	10^4^–10^7^	10^5^	the amplified virus in the mite gut
InjectLimit	0.1–100% of MiteVirusLoad	10%	a cap on the total number of viruses the mite injects
InjectSize	% of MiteVirusLoad	10%	a percentage taken if the virus population does not reach the InjectLimit
HighVirusLoad	10^5^–10^9^	10^7^	the amplified virus post-injection in pupae
	the following variables are embedded in the for-loop		
Varroabite	1–5%	3%	a sliding variable for the % of the virus population sampled during feeding
Varroainject	%	10%	a sliding variable for the % of virus injected into the next pupae

Briefly, the for-loop takes the 1000 viruses generated from Variants and PupaVirusLoad (table 2) and randomly samples them based on the BiteLimit. The selected variants are amplified in the mite using MiteVirusLoad as the maximum. An if-else loop then determines if the viruses in the mite have reached the limit imposed by InjectLimit, or not. If not, it takes a percentage defined as ‘injectSize’. InjectSize or InjectLimit then define the number of viruses passed to the next pupa, with a maximum amount defined outside the loop (100 000). A new pupa is generated with the virus variants DDD, VVV and VVD, totalling 1000 copies (PupaVirusLoad+Variants) and the capped MiteVirusLoad is added. This new mix is then amplified using HighVirusLoad. The resulting virus population reflects the virus amplified in the newly injected pupa and the low-level mixed population passed *per os*. This process repeats for 10 cycles of pupae before stopping. Each virus is calculated as a percentage of the total virus population and determined at the point where the amplified virus is mixed with the PupaVirusLoad in the new pupa. The full 10 cycle loop is repeated 10 times to give 10 populations, which are then used to create an average for each altered variable in figure 2.

## 3. Results

### (a) Virus replication and pathogenesis in drones

**Figure 3.**
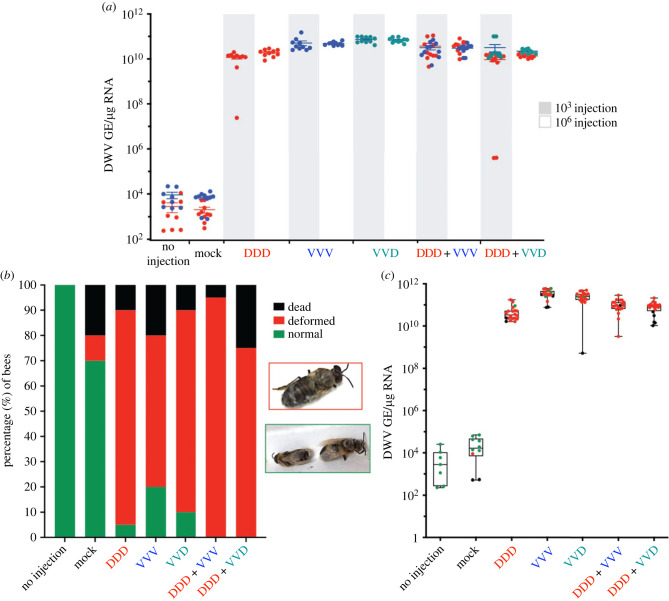
Quantified viral load of DWV variants in drone pupae. (*a*) RT-qPCR analysis of drones injected with DDD, VVD and VVV viruses individually or as co-infections mixes of the recombinant and either DDD or VVV, using two virus concentrations (distinguished by the white and grey bars) and incubated for 48 h. (*b*) Drone morbidity during infection with different DWV variants. Bees were assigned to one of three phenotype groups and the percentage of each is shown for single infections and co-infections. Each virus was injected into 20 drones, control groups consisted of 10 injected drones. The key on the right indicates how bees were scored with the pictures indicating the deformed and normal phenotypes. (*c*) Pupae injected with 10^2^ GE RNA of three DWV variants and left to eclose were quantified for their viral load after eclosion. Samples are coloured as per their phenotype at eclosion in (*b*).

The three major variants of DWV (Types A, B and a B/A recombinant) have all been shown to replicate similarly in (female) worker bee pupae and cause symptoms typically associated with DWV disease [21,27]. However relatively little is known about the replication and pathogenesis of DWV in drone pupae which have a longer developmental cycle. To examine this, age-matched drone pupae were injected with two concentrations (10^3^ and 10^6^ GE, selected as the lower and higher levels of mixed population infection levels [15,21]) of three DWV variants (DDD—Type A; VVV—Type B; VVD—recombinant) and incubated for 48 h. The resulting viral load, quantified by RT-qPCR, revealed all virus variants amplified to very high viral levels (greater than 10^9^ GE/*μ*g RNA) except for one of the DDD samples (figure 3*a*). Equimolar co-infections of DDD/VVV and DDD/VVD, which could be distinguished using type-specific qPCR primers, revealed that both viruses reached similarly high levels. However, two low-level DDD/VVD samples had considerably lower DDD levels (DDD approx. 10^5^ GE/μg RNA and VVD approx. 10^10^ GE/μg RNA).

Injected worker pupae all develop broadly equivalent viral loads, comparable to those recorded in drones in this study (figure 3*a*), but only approximately 80% have overt deformed wings upon eclosion. To examine whether morbidity differs in drones, white-eyed pupae were injected with 10^2^ GE/μg RNA of the three variants individually or with two co-infections (VVV/VVD, DDD/VVD) and left until eclosion (8–10 days). Figure 3*b* shows the percentage morbidity of each group, with emerging drones classified as dead, deformed or apparently normal with figure 3*c* showing the final viral level after eclosion. Despite all drones injected with a single variant having similarly high genome equivalents of DWV, 5–20% of the bees had normal wings, displaying no obvious symptoms of DWV infection. Interestingly, 100% of co-infected pupae had deformed wings upon eclosion (figure 3*b*/*c*).

### (b) Serial passage of DWV variants in drone pupae

As all variants produced similarly high genome equivalents by RT-qPCR as well as morbidity in drones, an experiment was designed to test the variants in competition during mixed infections across multiple generations of drone pupae by serial passage, broadly recapitulating mite-mediated virus transmission in successive generations of developing brood. Similar to observed bottlenecks in other vector-borne diseases like Dengue [24], we hypothesized that the volume of virus acquired by the mite during feeding could be key in shaping the population dynamics of DWV. ‘Mite-feeding’ and ‘re-injection’ was mimicked by preparing a crude virus extract, diluting it 100-fold and injecting a proportion into a pupa with low levels of endogenous DWV (figure 1). This was performed as a serial passage across 10 sets of drones using single virus injections and an inocula containing an equimolar mix of clonal DDD, VVV and VVD at the start. Virus levels in each successive passage was quantified by RT-qPCR (electronic supplementary material, figure S1) and subjected to next generation sequence analysis. electronic supplementary material, figure S1 indicates all variants reached similar GE across the ten generations of injections and moreover, virus levels continued to increase with each generation.

**Figure 4.**
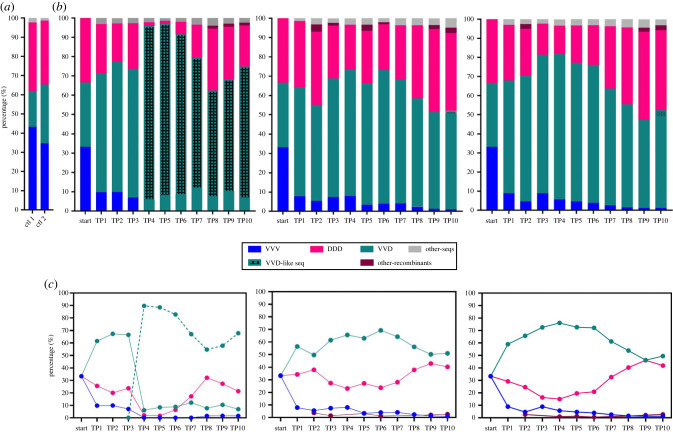
Analysis of changes in virus dominance over 10 passages in three generations of drone pupae. (*a*) Barplots show the percentage of each of the three variants in two control samples, one pre-PCR mix (ctl 1) and one post-PCR mix (ctl 2). (*b*) The percentage of each variant identified in individual drone pupae in 10 passages of three experimental sets, determined using ShoRAH analysis. The grey bar represents sequences below the error threshold used in ShoRAH (less than 1.5% of the population). (*c*) Line graphs illustrating the change in the dominant variant in each of the passages for the three datasets. The dashed line in dataset 1 represents the VVD-like variant identified.

Population changes upon passage of the mixed inocula were determined using ShoRAH to analyse a region of the viral genome which contained two introduced unique translationally silent restriction sites (SRS) for VVV/DDD and VVD (electronic supplementary material, figure S2). The resulting haplotypes produced were compiled and the percentage change in each variant over time was calculated (figure 4). In each of the three biological replicates examined, a recombinant form of DWV (VVD) became the dominant variant in the population across the ten generations of pupae. In set 1, in addition to the VVD recombinants used in the inocula, we detected the appearance, transmission and amplification of novel recombinants between the injected viruses in the inocula, or potentially with endogenous DWV. In all three replicates VVV was detected as the lowest percentage of the three variants and was lost completely in set 1 from time point (TP) 4 onwards. However, no viruses derived from the inocula, nor any *de novo* generated recombinants, replaced all other variants in any of the three biological replicates.

### (c) In silico modelling potential virus replication bottlenecks in a mite/bee/virus system

During serial passage no clonality within the 1500 bp window of the genome studied was observed despite the high virus loads transferred between bees. However, clonality has been reported in studies of natural virus transmission situations [12]. Similar to other virus/vector/host systems, the DWV population may undergo dynamic shifts in diversity and level depending on a range of factors [34,35]. These include virus acquisition by the mites, viral replication in the mite, virus levels achieved in the mite salivary glands and the amount of infectious virus injected as the mite feeds, together with the extensive subsequent replication in the host pupa post-injection.

Varroa feed on multiple pupae, moving from one cell to another for several cycles. This facilitates the spread of DWV and potentially acts as a bottleneck for virus replication, either through limited virus acquisition, or by being a site of selective replication of certain variants [23]. Using R studio, we developed an *in silico* model to investigate potential bottlenecks in the bee/mite/virus system at which the viral population could be significantly reshaped.

**Figure 5.**
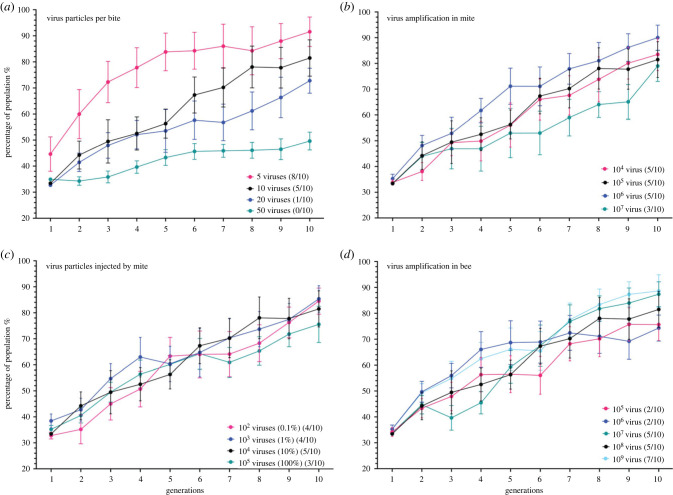
Four key variables modelled in the virus-mite-bee system. Modelling output for four stages of the cycle where the virus population may change, with the percentage that one variant makes up of the multi-variant population shown in the *y*-axis. (*a*) The virus particles acquired by the mite during feeding. (*b*) The amplification of the virus in the gut of the mite. (*c*) The virus particles injected into the pupa by the mite when it next feeds, as copy number and a percentage of the viruses in the mite gut. (*d*) The subsequent virus amplification in the parasitized pupa. The numbers in the schematic in figure 2 highlight the four stages of the model output shown in (*b*). The black line in each plot indicates the default value for that variable, with the other lines indicating changes to that parameter as stated in each key. Error bars indicate the SEM for the ten iterations of the model processed. The number of iterations (out of 10) which resulted in viral clonality (greater than 99% of the population) are shown in parentheses for each variable.

We reasoned that four key variables were (1) the number of infectious viruses ingested by *Varroa* feeding on a pupa, (2) the amount of virus replication in the mite, (3) the number of infectious viruses introduced to the next parasitised pupa and (4) the resulting amplification of the virus during replication in the pupa. The role of each was modelled using field-realistic values derived from published literature (table 1), typically on a sliding scale of 4/5 different values for each (table 2). Following ten repeated iterations of the model changes in the percentage of the dominant variant was measured in each passage for a particular level of each of the four key variables (figure 5).

Logically, the fewer virus variants ingested by the mite, the more likely the recipient pupa would amplify a clonal virus population. If only one virus was ingested or transmitted by the mite, or the donor pupa already carried a clonal virus population, only one virus would become amplified in the recipient pupa. We modelled the influence of the number of virions ingested over the range 5–50 and observed that ingestion of greater than 5 viruses resulted in clonality in ≤50% of the final pupae after repeating the *in silico* passage ten times. With only 5 viruses ingested by the mite, clonality was reached in 80% of cases (figure 5*a*).

We next modelled viral replication in the mite and virus injection into recipient pupae. Here, over the ranges tested, we found that neither variable dramatically altered the proportion of *in silico* passages that resulted in clonality, all of which were in the range 30–50% (figure 5*a*,*b*).

The final variable tested was the influence of virus amplification (range set between 10^5^ and 10^9^) in the recipient pupa on virus diversity. Studies have shown very marked levels of virus replication within 24–48 of inoculation in workers [21,27,36] and drones (this study). Essentially the model demonstrated that the more amplification of virus that occurred in pupae, assuming all viruses competed equally, the more chance the virus population would reach clonality, with amplification up to 10^9^ clonality was reached in 90% of cases (figure 5*d*). Given clonality was reached in some iterations of the model, but not all, the model recapitulates the stochastic processes that are observed in studies of virus diversity in the field and in laboratory-controlled experiments.

## 4. Discussion

Varroa destructor infestation and the consequent transmission and amplified replication of Deformed wing virus are strongly associated with overwintering colony losses. These losses, which regularly exceed 20% in surveys of beekeepers, and can exceed 50% in harsh or long winters, have a major impact on colony management and productivity [9,37–39]. DWV is an endemic virus, typically spread horizontally in the colony, however, the introduction of Varroa introduces a novel route of transmission, via direct inoculation of developing pupae or adult bees [40,41]. It is thought that this novel route allows the bypassing of the usual immune barriers in the host [42], with the resulting uncontrolled replication of the virus resulting in tissue damage and overt pathogenesis of the parasitized pupae.

It is known that Varroa preferentially parasitise drone cells in the colony [10], where the longer development cycle allows production of more progeny mites. However, it is unclear whether the replication kinetics of the virus, and consequent pathogenesis, is similar in drones and worker pupae; the majority of published studies have used worker pupae in experiments. We investigated the replication of DWV and the resulting morbidity of emerging drones when pupae were infected with the major variants of DWV, Type A, Type B and a B/A recombinant. Virus levels of all variants rapidly elevate in drones via injection, with levels reaching greater than 10^9^ GE/μg RNA within 48 h of injection regardless of the variant of DWV (figure 2*a*). The extent and proportion of emerging drones that exhibited overt developmental deformities was similar to that seen previously in worker bees, with greater than 80% of drones presenting with deformed wings when injected as white eyed pupae (figure 2*b*). As with earlier studies with worker brood [21,27], a proportion of the drone brood apparently developed without overt symptoms, but nevertheless exhibited highly elevated levels of DWV, indistinguishable from those in drones with wing deformities, or that died pre-eclosion (figure 2*c*). This indicates that it is not virus level alone which necessarily causes deformities. Workers with normal wings, but similarly high virus levels, are also observed in naturally mite infested colonies [15]. It is not clear if these workers perform any duties for the colony, such as feeding brood or foraging. If they are able to partially function like healthy workers they may transmit high levels of DWV via feeding/trophallaxis or potentially create spillover events by spreading it among other insects in shared ecosystems, such as via flowers [43]. If highly infected asymptomatic drones are functionally intact, they may be able to mate with queens and transmit high levels of virus via their sperm [44], which could consequently be transmitted vertically by the queen to her eggs [4]. This would allow DWV to bypass mite transmission and infect a colony via the queen.

Reports of DWV transmitted by Varroa have indicated that over time, as Varroa levels increase in the colony, the diversity of the DWV population reduces at the colony level [12]. However, analysis of individual workers carrying very high virus titres, or infected by injection, indicate that mixed populations of DWV variants often still occur when the titre is very high [13–15]. Here, we investigated how the virus population changed during ten serial passages in drone pupae, initially inoculated with an equimolar cocktail of three DWV variants. Although a similar pattern was observed in all three data sets, with a recombinant virus eventually dominating the population, none of the passages resulted in 100% domination of one variant by the final passage and all three variants could still be detected in two of the data sets (figure 3). As the analysis method looked at a 1500 bp window spanning the structural and non-structural proteins (where the silent restriction sites were located) we cannot state that one variant became clonal, as it is possible recombinants formed elsewhere in the genome during the serial passaging. Interestingly one replicate was dominated by a recombinant that did not contain the silent restriction site, but was otherwise identical to the injected recombinant VVD, but even this did not reach clonality within the 1500bp window in the timeframe of the experiment. These findings are similar to those of [19,20], where studies of multiple passages of DWV using wild-type stocks and mixed populations did not change significantly over time. Five generations of passaged Type A/B did not result in the selection of one variant over the other [20], suggesting factors independent of the injection process are influential in shaping the virus population, or that significantly more than 10 serial passages are required to reproducibly reach clonality. Although it is possible that the experimental injection of pupae poorly recapitulates the transmission of virus by the mite, it seems likely that there may well be multiple stages in the mite/bee/virus transmission which could shape the virus population [45].

When generating the model to examine changes in virus diversity over time, one bottleneck likely to shape the virus population was the number of viruses taken up by the mite during feeding [45]. Studies of other vector host systems have indicated this could be very low, with 5–40 copies of Dengue virus being transmitted by mosquitoes [24], and aphids imposing a 0.5–3.2 virion bottleneck on Potato Y virus [26]. Tick-based vector systems, like the transmission of Powassan virus by Ixodes sp, can result in slow, long-term changes in virus diversity, but the selective pressures which shape the population are similar to mosquitoes [46,47]. These studies were used as the basis for the low bite volume used in the model (table 2, figure 5). The lower the number of viruses consumed by the mite, the more likely the virus population reaches clonality, perhaps even irrespective of whether the viruses replicated in the mite gut or not before being passed to a pupa during feeding. Based on these findings it is likely that multiple variables need to be considered experimentally, including the antiviral immunity in the mite, subsequent replication in the mite, and the amounts of virus injested and transmitted by the mites, in order to recapitulate the bottleneck effects likely to occur when the mite is feeding on a pupa.

Different variants of DWV appear to have selective advantages in the mite, with evidence of Type B and recombinants thereof replicating in mites [21,22], with Type A variants transmitted in a non-propagative manner during feeding [23]. How much a particular variant is transmitted during mite salivation, or whether there is competition between variants in the salivary glands of mites, is unknown. These factors, combined with a limited number of viruses consumed by the mite during feeding, would likely rapidly shape the virus population at the colony level. Individual workers selected and screened could still carry mixed populations of DWV, at high viral loads, depending on the progress of the virus population in the colony and this may account for reports of high-GE mixed virus populations [13–15] . Recent reports have indicated a shift in the dominant variant of DWV in field samples, from Type A to Type B [15,48–52]. It seems likely that there may be bottleneck events that occur during virus ingestion or inoculation by the mite, compounded by selective amplification of Type B variants through replication, resulting in the apparent consequent landscape-scale dominance of Type B variants over Type A.

Despite the unknown variables in the model system, we believe it provides a valuable tool for determining virus changes in the mite/bee/virus system, and has indicated target areas for future research. These include quantifying virus levels, diversity and replication in mite salivary glands and accurately measuring the amount of DWV transmitted by the mite through salivation experiments using feed packet systems to further our understanding of how much virus is passed from mite to pupa. A fluorescent-tagged DWV stock [21,53] with a feed packet system could be used to determine where in the mite DWV localizes and replicates and how much of the ingested virus is then found in the salivary gland and transmitted further. Future virus passage work, in drones or workers, should focus on recapitulating the bottleneck events predicted to occur during mite feeding by diluting crude stocks to very low titres. Our previous studies involving direct virus inoculation have demonstrated extensive virus amplification in recipient pupae from as few as 10 infectious viruses [21]. It would therefore be interesting to conduct further virus passage studies, in drone or worker pupae, using very low titre mixed virus inocula which may better recapitulate the dynamics of virus transmission from mites to bees.’

## Data Availability

All of the NGS data files used in this paper are publicly available in the SRA (short read archive) of NCBI, which is accessible under BioProject ID: PRJNA888084. The data are provided in electronic supplementary material [54].
